# Conditionally immortalised equine skeletal muscle cell lines for *in vitro* analysis

**DOI:** 10.1016/j.bbrep.2022.101391

**Published:** 2022-12-05

**Authors:** Mary F. Rooney, Nuno G.B. Neto, Michael G. Monaghan, Emmeline W. Hill, Richard K. Porter

**Affiliations:** a School of Biochemistry and Immunology, Trinity Biomedical Sciences Institute (TBSI), Trinity College Dublin, 152-160, Pearse Street, Dublin 2, Ireland; b Trinity Centre for Biomedical Engineering, Trinity Biomedical Sciences Institute (TBSI), Trinity College Dublin, 152-160, Pearse Street, Dublin 2, Ireland; c Department of Mechanical, Manufacturing and Biomedical Engineering, Trinity College Dublin, Dublin 2, Ireland; d Plusvital Ltd., The Highline, Pottery Road, Dun Laoghaire, Co. Dublin, Ireland; e School of Agriculture and Food Science, University College Dublin, Belfield, Dublin 4, Ireland

**Keywords:** Equine, Skeletal muscle, Cell culture, Immortalised cells, Coenzyme Q_10_, Bioenergetics, Mitochondrial function, Myostatin, Thoroughbred horses

## Abstract

**Background:**

Thoroughbred racehorse performance is largely influenced by a major quantitative trait locus at the *myostatin* (*MSTN*) gene which determines aptitude for certain race distances due to a promoter region insertion mutation influencing functional phenotypes in skeletal muscle. To develop an *in vitro* system for functional experiments we established three novel equine skeletal muscle cell lines reflecting the variation in phenotype associated with *MSTN* genotype (CC/II, CT/IN and TT/NN for SNP g.66493737C > T/SINE insertion 227 bp polymorphism). Primary equine skeletal muscle myoblasts, isolated from Thoroughbred horse *gluteus medius*, were conditionally immortalised and evaluated to determine whether cell phenotype and metabolic function were comparable to functional characteristics previously reported for *ex vivo* skeletal muscle isolated from Thoroughbred horses with each genotype.

**Results:**

Primary myoblasts conditionally immortalised with the temperature sensitive SV40TtsA58 lentivirus vector successfully proliferated and could revert to their primary cell phenotype and differentiate into multinucleated myotubes. Skeletal muscle fibre type, *MSTN* gene expression, mitochondrial abundance, and mitochondrial function of the three *MSTN* genotype cell lines, were consistent with equivalent characterisation of *ex vivo* skeletal muscle samples with these genotypes. Furthermore, addition of coenzyme Q_10_ (CoQ_10_) to the cell lines improved mitochondrial function, an observation consistent with *ex vivo* skeletal muscle samples with these genotypes following supplementation with CoQ_10_ in the diet.

**Conclusions:**

The observation that the phenotypic characteristics and metabolic function of the cells lines are equivalent to *ex vivo* skeletal muscle indicates that this *in vitro* system will enable efficient and cost-effective analyses of equine skeletal muscle for a range of different applications including understanding metabolic function, testing of nutritional supplements, drug test development and gene doping test development. In the multi-billion-euro international Thoroughbred horse industry research advances in the biological function of skeletal muscle are likely to have considerable impact. Furthermore, this novel genotype-specific system may be adapted and applied to human biomedicine to improve understanding of the effects of myostatin in human physiology and medicine.

## Highlights

• Equine skeletal muscle cell lines developed to study equine muscle *in vitro*.
• Cell line phenotypes equivalent to *ex vivo* skeletal muscle.
• Provides an efficient, ethical, and cost-effective method to study equine muscle.

## 1 Background

The development of cell culture systems for functional studies reduces the need for *in vivo* experiments while allowing a broader study of cell physiology, disease progression and drug testing [1,2] and provides a cost-effective and ethically prudent approach [3,4]. Several immortalised mammalian tissue cell lines have been created, particularly as models mimicking cancer or disease (*e.g.* HeLa, human cervical cancer [5]; MCF-7, human breast cancer [6]; SH-SY5Y, human neuroblastoma [7]). There are fewer immortalised cell lines representing the wild-type or unaffected phenotype (*e.g.* C2C12, mouse skeletal muscle [8]; HEK-293, human embryonic kidney cells [9]), for these cell types, researchers frequently use primary cells or tissue when assessing normal cell function. However, working *in vivo*, with *ex vivo* tissue samples and working with primary cells, all have their limitations; working *in vivo* or with *ex vivo* tissue samples can have logistical challenges, particularly in the field of livestock and large animal research. Thoroughbred horses, for instance, are large, valuable animals who by the nature of their purpose are either in active race training or breeding and are most commonly privately-owned rather than maintained in research herds and therefore regular tissue sampling is challenging. Primary cells, while advantageous due to their close representation of *in vivo* models, will generally only grow in culture for a limited number of divisions before cell cycle arrest (cellular senescence) [[10], [11], [12]], meaning further tissue sampling would be required to maintain a cell stock. Although some primary cell lines have been capable of extended growth and subsequent use as cell models for functional analysis *in vitro*, due to spontaneous immortalisation [[13], [14], [15]], this is not a common occurrence. Immortal cell lines alleviate these limitations by providing a means of prolonged cell growth enabling continuous experimental research. To our knowledge, there is no commercially available immortal equine skeletal muscle cell line, although several laboratories have developed equine fibroblast and skeletal muscle cell lines tailored to specific experimental requirements [[16], [17], [18], [19]].

The Thoroughbred horse industry is a long established highly economically valuable international industry. Novel techniques or experimental assessments that can further research in Thoroughbred physiology for the improved health and wellbeing of these animals will have strong economic bearing. Therefore, we considered that the development of an *in vitro* immortalised equine cell line would provide considerable potential for refining the understanding of equine skeletal muscle metabolism and for the assessment of nutritional supplements. Research into the benefits of nutritional supplements has been somewhat limited to-date [[20], [21], [22]], potentially due to the cost, and the logistical and ethical constraints of *in vivo* and *ex vivo* analysis in these animals. There are also many misconceptions, due to a lack of detailed information on some supplements, meaning consumers are often unaware of the specific benefits and/or the actual efficacy of the supplement they are purchasing [[23], [24], [25]]. This is also the case for humans, where similarly, nutrient supplementation is prevalent, but the research is relatively limited [[26], [27], [28]].

The development of an *in vitro* model provides a cost-effective and practical system for *in vitro* testing of the potential benefits of supplements. This could have a huge impact on the supplement industry, by providing scientific evidence on the benefits and the efficiency of nutrients available to consumers. An advantage of the *in vitro* system will be analysis of specific ingredients contained in multi-nutrient supplements, therefore potentially reducing costs by removing ineffective or unnecessary compounds and in turn reducing the unnecessary substances consumed by the end user. Increasing research efforts on equine supplements will provide consumers with the information they need to make informed choices before using a supplement. Similar research could be applied to supplements for human consumption, either as initial testing in these equine cells with follow up *in vivo* studies or specific experimentation on a smaller scale with human primary muscle cells.

*In vitro* systems can have several other beneficial applications such a tool in gene editing research and for testing the effects of banned substances. Drug doping in the world of equine sports and in the realm of sports in general is constantly evolving and with each new method of doping comes the requirement to find a method of detection. Currently the process of doping testing in horses is complex [29], with little harmonisation across the governing bodies worldwide. Finding ways to cope with the evolving doping strategies and streamlining the time-consuming process of testing are highly desirable. Some suggest the use of metabolomics as an answer, but there are many limitations still to be addressed before this could become an efficient option [30]. Another possible option would be to test for biomarkers of drug doping, alleviating the need for the time-consuming process of testing for each illicit substance [[31], [32], [33]]. This would first require identification of such biomarkers. The functional genomic response to doping agents could be examined *in vitro* to identify biomarkers that could then be used as indicators for exposure to a particular agent. In addition, the metabolic, gene expression, immunological, signal transduction *etc.* response to doping substances could also be assessed *in vitro* to gain further detailed insight into the specific cellular impacts of doping agents.

Further sophisticated methods of doping by manipulating the inherited genome of an individual have become possible through advancements in gene therapy. Although not yet commonplace, research in the field of gene therapy is constantly improving [[34], [35], [36], [37]] and with every advancement in genetic methods intended for hereditary disease therapies, comes further opportunities for exploiting the technology for doping strategies [[38], [39], [40], [41], [42]]. Therefore, developing methodologies to combat this is necessary [[43], [44], [45]]. Identifying probable targets for gene doping would be the first port of call in combating gene doping [46] followed by studying the gene expression profiles of equine skeletal muscle cells in response to various doping strategies. ‘Normal’ expression profiles in the published literature could be utilized for additional comparisons; such as *MSTN* which has been extensively researched, with gene expression data available for various exercise conditions [47] and is a prominent feature in the *in vitro* cell culture system discussed in this article. An *in vitro* equine muscle system would provide an excellent model for optimisation and analysis of these gene doping methods, provide a system to study the gene expression profiles resulting from these doping strategies [48] and could ultimately aid the development of suitable gene doping detection methods.

Furthermore, all of the aforementioned applications could be adapted for and applied to human skeletal muscle, to improve understanding of human muscle phenotypes and enable improvement of drug doping testing, supplement research and gene doping in human skeletal muscle.

Therefore, here we describe the establishment of novel equine skeletal muscle cell lines that may be used to investigate functional phenotypic variation resulting from genetic variation at the economically important myostatin (*MSTN*) locus. Horses with different *MSTN* genotypes, assigned as CC/II, CT/IN and TT/NN for the combined SNP g.66493737C > T/SINE insertion 227 bp polymorphisms [49] have variable metabolic and functional phenotypes [47,[49], [50], [51], [52], [53], [54], [55], [56]] that impact suitability to perform as a sprinter, middle-distance or long distance (‘endurance’) racehorse. One of the principle phenotypic differences among the genotypes is a marked variation in skeletal muscle fibre type proportion and lower *MSTN* gene expression in the (CC/II) genotype [55,57,58]. We also previously reported that CoQ_10_, measured indirectly via electron transport chain complex I + III activity, in skeletal muscle of ‘endurance’ (TT/NN) genotype horses was significantly lower than that of the ‘sprint’ (CC/II) genotype, but observed in an ‘add-back’ experiment that addition of exogenous CoQ_10_ to *ex vivo* skeletal muscle samples from ‘endurance’ (TT/NN) genotype horses improved mitochondrial performance in that tissue [55]. This finding was mirrored in an *in vivo* supplementation trial in which oral dietary CoQ_10_ supplementation of Thoroughbred horses increased complex I + III activity 65% after a 9-week supplementation period [59].

Here, we describe the creation of equine skeletal muscle cell lines with the three distinct genotypes. Using these cell lines, we characterise the *in vitro* metabolic phenotypes of the *MSTN* genotype cell lines to assess the feasibility and accuracy of immortalised cell lines for ongoing experiments and establish an efficient *in vitro* module for equine skeletal muscle. An *in vitro* cell line of this kind, particularly with its comparative genetic attribute, will enable a wide range of analyses in skeletal muscle research which could have considerable impact on the field of equine skeletal muscle research and provide economic benefit in this multi-billion-euro international industry. This cell model could also act as a progenitor to a similar human skeletal muscle approach which would be valuable to the human skeletal muscle research field.

To our knowledge this is the first description of establishing normal/wild-type cell lines that are differentiated on the basis of genotype. Several cell lines have been established from mouse specimens where genes have been knocked-out [60,61], rather than developed with ‘naturally occurring’ polymorphic genotypic differences. Therefore, using this system for *in vitro* comparative genetic analysis is a unique approach that will allow for improved understanding of the functional effects of *MSTN* genetic variation that is not feasible *in vivo*.

## 2 Methods

### 2.1 Materials

Plasticware and other single-use consumables were obtained from Cruinn Ltd. unless stated otherwise. Cell culture media, fetal bovine serum (FBS), horse serum, phosphate buffered saline (PBS), TrypLE express dissociation agent, penicillin/streptomycin, puromycin, ProLong antifade gold mounting medium and anti-mouse alexaflour secondary antibody were from Biosciences Ltd. Polybrene transfection reagent was from Merck Millipore. Lentiviral vector for immortalisation and GFP control vector were from ABM (Germany). Slides and coverslips for immunofluorescence were from Fisher Scientific and VWR, respectively. Anti-desmin antibody (Cat# 550626) was from BD Biosciences, anti-sarcomeric myosin antibody (Cat# MAB4470) was from R&D systems and Hoechst 33342 stain was from Life Technologies. All other chemicals/reagents were from Sigma-Aldrich, unless stated otherwise.

### 2.2 C2C12 and 3T3-L1 cell culture

C2C12(ATCC® CRL-1772) cells, an immortal mouse derived adherent muscle myoblast cell line [8,62] and 3T3-L1 (ATCC® CL-173) cells, an adherent pre-adipocyte fibroblast cell line [63,64] were used as myogenic and non-myogenic controls, respectively, for immunostaining. Both cell lines were maintained in Dulbecco's Modified Eagle's Medium (DMEM) GlutaMAX cell culture medium (Gibco) (high glucose, with sodium pyruvate) supplemented with 10% (v/v) FBS (Invitrogen) and penicillin-streptomycin (50 U/ml and 50 μg/ml) (Gibco). Cells were grown at 37 °C in a humidified environment with 5% CO_2_. Cells were passaged at least twice weekly depending on their levels of confluency using TrypLE express (Gibco) dissociation reagent. C2C12 myoblast cells differentiated into myotubes upon confluency and differentiation was further encouraged by the reduction of serum concentration (to 2% horse serum) in the media. C2C12and 3T3-L1 cells were routinely tested for mycoplasma infections as per the method described by Young et al. [65].

### 2.3 Animals and sample collection

Skeletal muscle myoblasts can be isolated from cadaver tissue; once extracted, placed in the appropriate solution, and transported to the laboratory in a timely fashion for the isolation procedure; providing a sufficient and the most ethical method of procuring tissue for this study. In consideration of this, skeletal muscle tissue samples were acquired, with permission, from an Irish abattoir. The samples used in the study were extracted immediately post-mortem from three horses which were euthanized for non-research purposes, therefore Health Products Regulatory Authority (HPRA) approval was not required. Veterinary records described no previous muscle related veterinary conditions in the horses sampled. Samples were taken from the *gluteus medius* skeletal muscle by scalpel dissection at a depth of approximately 4–8 cm. All three horses were Thoroughbreds, aged 14 [male], 10 [female] and 25 [male] years of age and their *MSTN* genotypes were CC/II, CT/IN and TT/NN respectively (SNP g.66493737C > T/SINE insertion 227 bp).

### 2.4 *MSTN* genotyping

DNA was extracted from hair samples and genotyped for the *MSTN* SNP g.66493737C > T [66] and the *MSTN* SINE insertion 227 bp polymorphism [54]. Primary and immortalised cell samples were similarly genotyped at various stages in the immortalisation process to confirm cross-contamination of cell lines had not occurred.

### 2.5 Equine skeletal muscle myoblast isolation and culture

Skeletal muscle tissue samples were placed directly into ice-cold supplemented cell culture media (DMEM GlutaMax, high glucose, with sodium pyruvate (Gibco) supplemented with 20% FBS (Invitrogen), 10% horse serum (HS) (HyClone) and penicillin-streptomycin (50 U/ml and 50 μg/ml) (Gibco)) immediately post-extraction. Samples were transported on ice and stored at 4 °C for approximately 16 h prior to primary myoblast cell isolation.

To isolate myoblast cells, the muscle sample (approximately 1 g) was washed with supplemented DMEM in a Petri dish, any tendon, fat, vessels or connective tissue was carefully removed, and the tissue sample was sliced into very small fragments. The tissue fragments were transferred to a 50 ml falcon tube and allowed to settle to the bottom at which point the supernatant was aspirated and discarded. The muscle fragments were resuspended in a 0.1% pronase solution in media and incubated at 37 °C for 60 min with intermittent agitation every 15 min. After 1 h, an equal volume of media was added to the tube to stop the reaction. The muscle fragments were further dissociated using vigorous trituration. The sample was repeatedly pipetted 10 times with a 25 ml pipette, followed by 10 goes with a 10 ml pipette. The sample was subsequently centrifuged at 400 g for 3 min to pellet the debris. The supernatant (containing the extracted cells) was poured through a 40 μm cell strainer, which was then washed with fresh media, followed by centrifugation at 1000 g for 10 min to pellet the cells. The cell pellet was resuspended in a small volume of media and seeded in a gelatin (2%)-coated T25 flask in a ‘pre-plating’ step for 1 h, to encourage removal of fibroblasts. After 1 h the cells in suspension were removed and re-seeded in gelatin (2%)-coated culture dishes as appropriating depending on cell volume. Cell cultures were left undisturbed at 37 °C for 3 days. After 3 days the media was changed very gently, and the cells were continued to be monitored daily. First passage of cells usually took place between 7 and 10 days after isolation. At first passage cells were pre-plated as previous for 20 min before being re-seeded into a fresh gelatin (2%)-coated flask. Once a substantial population of cells had been obtained at passage 3–4 cell stocks were frozen and remaining cells were plated for immortalisation or immunostaining.

### 2.6 Conditional immortalisation of equine myoblasts

This immortalisation protocol was adapted from a variety of publications [17,[67], [68], [69], [70]] to suit the parameters of this project and the optimal conditions for these cells.

Isolated primary myoblasts (passage 3–8 post isolation) were seeded at 5 x 10^4^ cells per well of a 6-well plate and cultured for 24–48 h until at approximately 70% confluency. At this point the media was removed and replaced with a 1 ml mixture of complete media supplemented with polybrene at a concentration of 8 μl/ml. The optimised multiplicity of infection (MOI) volume for the temperature-sensitive simian vacuolating virus 40 large T antigen (SV40TtsA58 lentivirus) vector (MOI5 for 5 x 10^4^ cells; 250 μl of 1 × 10^6^ IU/ml)) was added to each treatment well. The same amount of GFP control vector was added to a separate set of wells and at least one well was left untransduced as ‘positive’ and ‘negative’ controls, respectively. Cells were incubated at 33 °C (temperature sensitive vector allows immortal growth at 33 °C) for 24 h before the viral supernatant was removed and replaced with fresh media. Cells were incubated for a further 24 h at 33 °C at which point they were treated with 2 μg/ml of the selection antibiotic puromycin. Cells were cultured at 33 °C, passaging as necessary, for 7–14 days, at which point successful immortalisation of transfected cells could be concluded based on efficient proliferation in the presence of puromycin, compared to puromycin induced death of untransfected control cells. Positive cell colonies that had survived the antibiotic selection were selected from the cell population using cloning rings and expanded by seeding and culturing in 24-well plate wells and then further in T25 cm^2^ flasks. All clones were immunostained and imaged as per the methods described later in this manuscript (additional file 2). The cell clone chosen for each genotype was expanded further and stocks were frozen down and stored in liquid nitrogen vapour for future use. Immortalised cells were capable of growing in culture for numerous passages at 33 °C. Incubating the cells at 37 °C resulted in the cells becoming ‘primary-like’ and capable of terminal differentiation.

GFP control cell samples were visualised under bright field and fluorescence microscope to assess transfection efficiency in these cells (additional file 1, Supplemental Fig. 1A). Immunoblotting and PCR (as per methods in additional file 4) were also used to confirm the presence of SV40T in the immortalised cell samples (additional file 1, Supplemental Figs. 1B and 1C).

### 2.7 Immunostaining and confocal imaging

Cells (primary, immortalised, ‘primary-like’ and differentiated) were immunostained and imaged by confocal microscopy as per the methods described herewith. Cells were seeded at 2 x 10^4^ cells per well of a 24-well plate on inserted 12 mm circular glass coverslips and were incubated at 33 °C (immortalised) or 37 °C (primary, ‘primary-like’, differentiated) for 2 days until approximately 90% confluent. At this point media on the cells destined for differentiation was changed to the reduced serum (2% HS) media and the cells were cultured for a further 5–10 days until the cells terminally differentiated into myotubes.

To fix the cell cultures, media was removed, cells were washed and 200 μl of ice-cold acetone:methanol (1:1) fixative solution was added to each well and the cells were fixed for 10 min at room temperature. The fixative solution was removed carefully, and the well was washed three times with tris-buffered saline (150 mM NaCl, 20 mM Tris-HCl, pH 7.6) (TBS). To block nonspecific antibody binding, 500 μl of blocking solution (TBS supplemented with 5% BSA) was added to each well and the plate was incubated at 4 °C overnight. Following overnight incubation at 4 °C the plates were returned to room temperature and washed three times with TBS prior to antibody staining. Primary antibodies were diluted in blocking solution at 1:100 and 1:250 dilution of desmin (BD) and sarcomeric myosin (R&D/Bio-techne), respectively and 150 μl of appropriate antibody solution was added to the appropriate well. Cells were incubated with primary antibodies at room temperature with gentle swirling for 3 h. Subsequently, wells were washed with TBS three times and then incubated with goat anti-mouse AlexaFluor 488 secondary antibody (thermo-fisher) at 1:1000 dilution in blocking solution for 1 h at room temperature. The secondary antibody solution was removed, and the cells were washed with TBS as previous. For nuclear visualization 100 μl of Hoechst solution (1 μg/ml diluted in TBS) was incubated with the cells for 30 min at room temperature. Cells were washed once again prior to the coverslip being mounted on to a glass slide using 1 drop of ProLong Antifade mounting medium. Slides were kept in the dark for 24 h at room temperature, then sealed and imaged using confocal microscopy. A Leica SP8 scanning confocal microscope with a 488 laser (max excitation 490, max emission 525) at 20X zoom was used to image slides.

### 2.8 Enzyme assays

Citrate synthase enzyme activity was used as a marker of mitochondrial abundance in the skeletal muscle cells. CoQ_10_ content was measured indirectly by spectrophotometric combined complex I + III assay. Enzyme activity assays were performed at 30 °C on a Libra S12 spectrophotometer (Biochrom Ltd., Cambridge, UK) with absorbance changes measured using an attached chart recorder. The activity of each enzyme was measured in triplicate on the same homogenate for each sample.

#### 2.8.1 Preparation of cell homogenates

Equine skeletal muscle myoblasts were grown and differentiated as described earlier in methods section. Equal numbers of cells were pelleted and washed with PBS before being resuspended in 100 μl hypotonic potassium phosphate buffer (25 mM K_2_PO_4_, 5 mM MgCl_2_). Each sample was subjected to three freeze thaw cycles in liquid nitrogen to disrupt biological membranes. An aliquot of each sample was used to perform protein determination using the bicinchoninic acid assay as described by Smith et al. [71] and the rest was used for enzyme activity assays.

#### 2.8.2 Citrate synthase activity assay

Citrate synthase enzyme activity (a measure of mitochondrial abundance) was measured spectrophotometrically by a coloured coupled reaction, using a method adapted from Srere [72]. The activity of citrate synthase was determined by monitoring the rate of production of thionitrobenzoic acid at a wavelength of 412 nm. Skeletal muscle cell homogenate was incubated in a 1 ml cuvette with tris buffer (0.2 M, pH 8.1) with reaction components added: 5,5′-dithiobis-(2-nitrobenzoic acid) (0.1 mM), acetyl coenzyme A (0.3 mM) and Triton X (0.1%). A blank rate was measured for 2 min before oxaloacetate (0.5 mM) was added to initiate the reaction and an increase in absorbance was monitored for 3 min. Specific enzyme activity was expressed as pmol/min/mg of muscle protein using the molar extinction coefficient 13,600 L/mol/cm for citrate synthase at 412 nm.

#### 2.8.3 NADH cytochrome *c* oxidoreductase (complex I + III) activity assay

The activity of NADH cytochrome *c* oxidoreductase (Complex I + III, an indirect measure of CoQ_10_) was determined by monitoring the reduction of cytochrome *c* at 550 nm, as per the method described by Powers et al. [73]. Cell homogenates were incubated in distilled H_2_O in a 1 ml cuvette to allow osmotic shock to occur. After 2 min incubation, the reaction components were added: potassium phosphate pH 7.5 (50 mM), oxidised cytochrome *c* (50 μM), KCN (0.3 mM), and fatty-acid free BSA (1 mg/ml); a blank rate was measured for 2 min. NADH (0.2 mM) was then added to initiate the reaction and an increase in absorbance was monitored for 3 min. Following this, rotenone (10 μM) was added and the rate was monitored for a further 2 min. Complex I + III combined specific activity was taken as the rotenone-sensitive activity determined by subtracting the rotenone-resistant activity from the total activity. Specific enzyme activity for complex I + III was expressed as pmol/min/mg of muscle protein using the molar extinction coefficient 18,500 L/mol/cm for reduced cytochrome *c* at 550 nm. Complex I + III activity was subsequently expressed as a ratio to citrate synthase activity to account for the mitochondrial enrichment of the cells.

### 2.9 Gene expression

Total RNA was isolated from skeletal muscle cell samples using Qiazol reagent (Qiagen) and was isolated and purified using RNeasy Plus Universal kit (Qiagen) as per the manufacturer's instructions. Equal amounts of RNA were reverse transcribed into cDNA using the High-Capacity cDNA Reverse Transcription Kit (Applied Biosystems) as per manufacturer's instructions. Reverse transcription was performed by heating the reaction mix at 25 °C for 10 min followed by 37 °C for 120 min and 85 °C for 5 min. The resulting cDNA was then diluted with nuclease free water and used for real-time qPCR. Careful attention was paid to avoid PCR contamination and no false-positives were observed in negative controls. Biosystems 7500 Fast Real-Time PCR System and SYBR green reagents were used to measure mRNA in equine skeletal muscle tissue. Specific primers and qPCR conditions were as previously described for *MSTN*; Rooney et al. [54] and for *HPRT*, *MYH7*, *MYH2*, *MYH1*; Rooney et al. [55]. All reactions were run in at least duplicate. SDS 1.9.1 software (Applied Biosystems) was used to analyse the amplification curves and these curves were used to determine the relative mRNA expression of each gene. The expression of each gene was normalised to the expression of *HPRT* using the ΔΔCt method. Myosin heavy chain (MHC) isoform gene expression (*MYH7*, *MYH2* and *MYH1*) was subsequently expressed as a percentage of total MHC gene expression.

### 2.10 Cellular respiration using seahorse XFp analyser

Cellular oxygen consumption rates (OCR) were determined by Seahorse XFp Extracellular Flux analyser (Agilent Technologies) with 10,000 cells per well. Appropriate volumes of the mitochondrial inhibitors/uncouplers were added to the Seahorse XFp cartridge prior to experimentation and were introduced automatically by the machine in a sequential manner during the experiment. The following final concentrations were used; oligomycin 1 μM, FCCP 1 μM and antimycin A (AA)/rotenone 0.5 μM.

### 2.11 Fluorescence lifetime imaging microscopy (FLIM) of NAD(P)H

Immortalised equine skeletal muscle myoblasts were grown as per the usual methods in 35 mm petri dishes and at confluence were used for fluorescence lifetime imaging microscopy (FLIM) of NAD(P)H. FLIM was performed on a custom multiphoton system. A titanium:sapphire laser (Chameleon, Coherent) was used for multiphoton excitation on an upright (Olympus BX61WI) laser scanning microscope coupled with a water-immersion 25x objective (Olympus, 1.05NA). Two-photon excitation of NAD(P)H was performed using 760 nm as excitation wavelength. A 455/90 nm bandpass filter was used to isolate NAD(P)H fluorescence signal. Images (512x512 pixel) were acquired with a pixel dwell time of 3.81 μs and 30-s collection time. A PicoHarp 300 TCSPC system operating in the time-tagged mode coupled with a PMA hybrid detector (PicoQuanT GmbH, Germany) was used for fluorescence decay measurements yielding 256 time bins per pixel. The samples were placed on an incubating microscope stage at 37 °C and for each individual sample, at least three separate images from different areas of the dish were acquired.

The fluorescence images and decay curves for NAD(P)H were obtained and the overall decay curve was generated by the contribution of all pixels and was fitted with a double exponential decay curve, for GFP and NAD(P)H, respectively (Eq. (1))

(1) $$I\left(t\right)=\alpha_{1}e^{-\frac{t}{\tau_{1}}}+\alpha_{2}e^{-\frac{t}{\tau_{2}}}+c$$

*I(t)* corresponds to the fluorescence intensity measured at time *t* after laser excitation; *α*_*1*_ and *α*_*2*_ represents the fraction of the overall signal comprise of a short – free NADH and long lifetime – protein bound NADH component, respectively. *τ*_*1*_ and *τ*_*2*_ are the short and long lifetime components, respectively; *c* corresponds to background light.

X^2^ value is calculated to evaluate the goodness of multiexponential fit to the raw fluorescence decay data. In this study all the values with X^2^ < 1.3 were considered good fits.

The average lifetime (*τ*_avg_) of NAD(P)H for each pixel is calculated by a weighted average of both free and bound lifetime contributions (Eq. (2)).

(2) $$\tau_{avg}=\frac{\left(\alpha_{1}\times \tau_{1}\right)+\left(\alpha_{2}\times \tau_{2}\right)}{\left(\alpha_{1}+\alpha_{2}\right)}$$

The optical redox ratio (ORR) was calculated using equation (2):

$$ORR=\frac{{FAD}^{+}}{NAD\left(P\right)H}$$

With NAD(P)H being the total fluorescence intensity acquired at the 455–490 nm and FAD^+^ the total fluorescence intensity acquired at 502–547 nm. The intensity of both images at different channels was obtained by conserving the same region of interest (ROI) in the Symphotime® software.

### 2.12 Statistical analysis

Statistical analyses were performed using Prism (GraphPad Software, California, USA). Mean values were compared using a one-way or two-way ANOVA as appropriate, with a Bonferroni multiple comparison post-test, p-values where shown indicate significance (* = p ≤ 0.05, ** = p ≤ 0.01, *** = p ≤ 0.001), with results expressed as mean ± SEM unless otherwise indicated.

## 3 Results and discussion

### 3.1 Isolation and conditional immortalisation of equine skeletal muscle myoblasts

Obtaining fresh equine skeletal muscle biopsy specimens regularly for functional assays poses many limitations in sample collection which we sought to circumvent by establishing a series of conditionally immortalised equine skeletal muscle cell lines. Several successful immortalisations of skeletal muscle cells from other species such as human, mouse, pig and other mammals [8,70,[74], [75], [76], [77], [78], [79]] exist and successful immortalisation of equine skeletal muscle cells has previously been reported [17]. Here, the aim was to establish equine skeletal muscle cell lines exhibiting variable functional phenotypes associated with natural genetic variation in the population of interest, that would facilitate assessment of functional assays relevant to the variable phenotypes.

Therefore, we established three working immortal equine skeletal muscle cell lines that are distinguishable on the basis of genotype for the most economically important locus in the horse racing and breeding industry. Primary equine skeletal muscle myoblasts were isolated from three *MSTN* genotype Thoroughbred horses (CC/II, CT/IN and TT/NN for SNP g.66493737C > T/SINE insertion 227 bp polymorphism) and conditionally immortalised by transfecting with the SV40TtsA58 lentivirus vector. The SV40TtsA58 vector contains a temperature sensitive region (tsA58 gene, located between 3138 and 5264bp), with an alanine-to-valine mutation at amino acid 438, active only at 33 °C. When cells are cultured at this temperature, they grow immortally. When cultured at 37 °C they return to a ‘primary-like’ condition and are capable of terminal differentiation [[80], [81], [82]]. The general transfection efficiency of these cells was assessed using a green fluorescent protein (GFP) control vector and successful transfection specifically with the SV40TtsA58 vector was confirmed by detection of SV40T DNA and protein in cell samples (additional file 1).

**Fig. 1:**
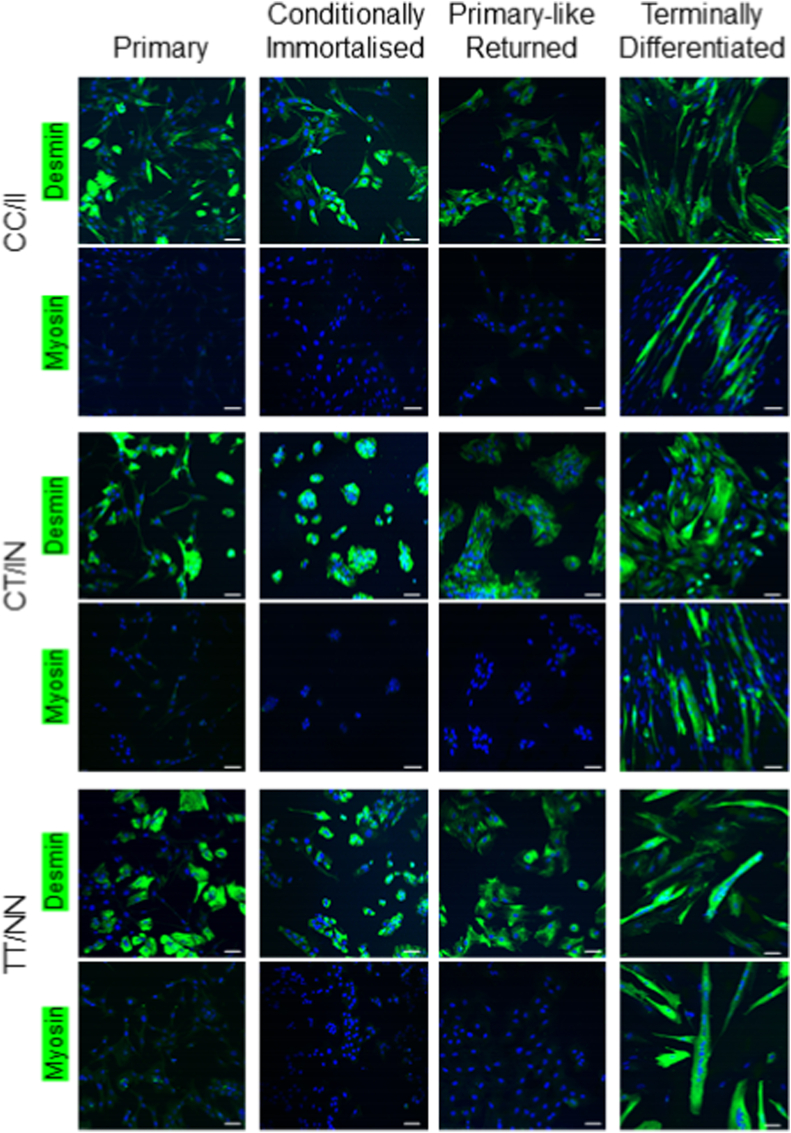
**Desmin and sarcomeric myosin expression in equine skeletal muscle cells:** Immunofluorescence images of Thoroughbred skeletal muscle; primary, conditionally immortalised, and primary-like myoblasts and terminally differentiated myotubes from three *MSTN* (CC/II, CT/IN, TT/NN) genotypes. Green, desmin (myogenic marker)/sarcomeric myosin (myosin) (differentiation marker); blue, nuclei. Images were take using a Leica SP8 confocal microscope at 20X. Scale bar (white) indicates 50 μM. Each image is representative of at least 3 independent experiments. (For interpretation of the references to colour in this figure legend, the reader is referred to the Web version of this article.)

After successful transfection, antibiotic and clonal selection was employed to isolate several cell clones for each genotype. We identified, selected, and expanded a single clone for each genotype. Through expansion of these cell lines, we confirmed that the cells could proliferate prosperously, and confirmed via immunostaining that these cell lines are of myogenic phenotype with positive expression of desmin (muscle-specific, type III intermediate filament) and confirmed that the cells could be terminally differentiated into multinucleated myotubes, using immunostaining for sarcomeric myosin (muscle-specific, involved in the coordination of skeletal muscle development) (Fig. 1). Immunostaining was performed on the cells at each stage; primary myoblasts, conditionally immortalised myoblasts, immortalised myoblasts reverted to ‘primary-like’ conditions and immortalised ‘primary-like’ terminally differentiated myotubes. 3T3-L1 and C2C12 cells were imaged as a negative- and positive-myogenic control, respectively (additional file 2).

### 3.2 *Fibre type proportions,* MSTN *gene expression and mitochondrial abundance of immortal cell lines are comparable to ex vivo skeletal muscle*

Having established that the immortalised equine skeletal muscle cell lines were capable of terminal differentiation when returned to ‘primary-like’ conditions, we characterised the phenotypic and metabolic status of each genotype, in order to validate and compare with characteristics previously obtained for *ex vivo* skeletal muscle tissue of the same genotypes [55,57].

**Fig. 2:**
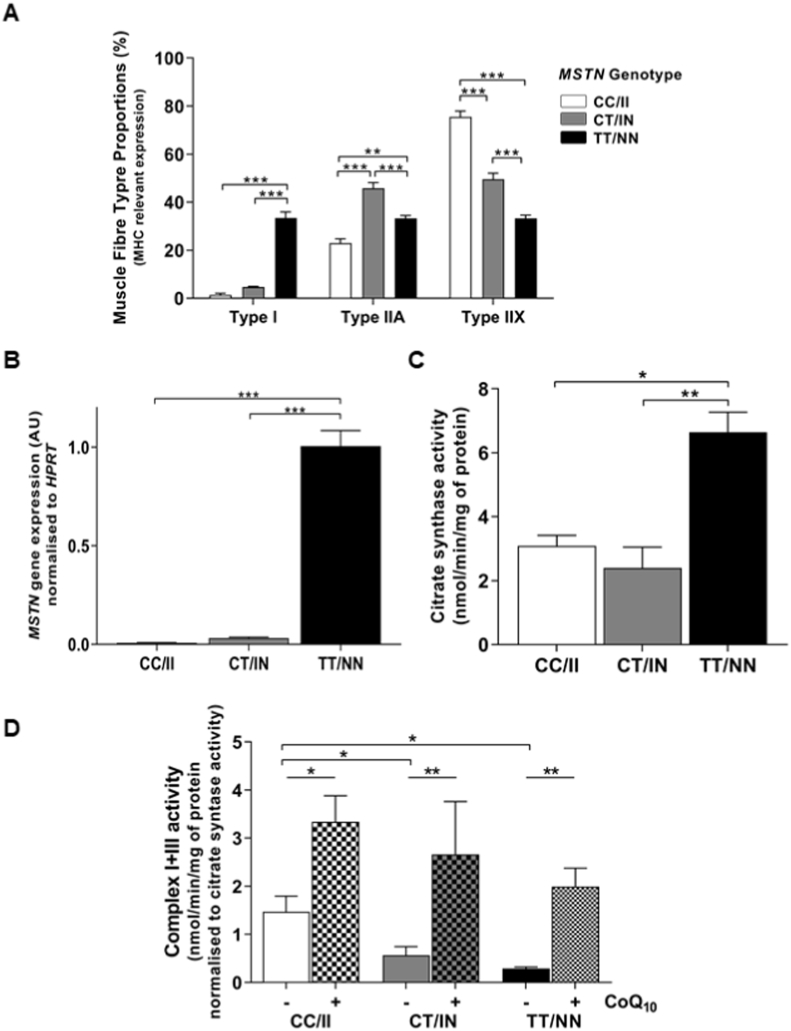
**Fibre proportions, *MSTN* gene expression, Mitochondrial abundance and CoQ**_**10**_**content of immortalised equine myotubes:** (A–D) Phenotypes compared among three *MSTN* genotype cell lines, CC/II (white); CT/IN (grey) and TT/NN (black). (A) qPCR was used to measure gene expression levels of: *MYH7*, *MYH2* and *MYH1* inferring MHC isoforms and interpreted as Type I, Type IIA and Type IIX fibres, respectively, expressed as relative percentage of total MHC gene expression. (B) *MSTN* gene expression was measured by qPCR, normalised to *HPRT* using the ΔΔCt method. (C) Mitochondrial abundance determined by the activity of citrate synthase measured spectrophotometrically, expressed as nmol/min/mg of muscle protein. (D) NADH cytochrome *c* oxidoreductase (Complex I + III) activity, as an indirect measure of CoQ (ubiquinone), measured spectrophotometrically and expressed as nmol/min/mg of muscle protein, normalised to citrate synthase activity. – (plain bars) and + CoQ_10_ (5 μM for 24 h) treated (dotted bars) samples, were analysed. (A–D) Results presented with mean ± SEM (N = 3 per genotype, performed in triplicate technical replicates), p-values where shown indicate significance as measured by; a two-way (A, D) or one-way (B, C) ANOVA with a Tukeys multiple comparisons test * = p ≤ 0.05, ** = p ≤ 0.01, *** = p ≤ 0.001.

Previously using muscle biopsy samples [55], we observed a significant difference in muscle fibre type proportions among the three genotypes [55]. The expression of three genes; *MYH7*, *MYH2* and *MYH1,* which are primarily expressed in Type I, Type IIA and Type IIX muscle fibres respectively [80,83], were quantified (Fig. 2A). In agreement with *ex vivo* observations, the TT/NN cell line had higher *MYH7* gene expression and indicating it contained a higher proportion of type I fibres compared to the CC/II (p ≤ 0.001) and CT/IN (p ≤ 0.001) cell lines. The opposite profile was observed for expression of *MYH1*, with the CC/II cell line having a higher proportion of type IIX fibres compared to the CT/IN (p ≤ 0.001) and TT/NN (p ≤ 0.001) cell lines. The CT/II cell line had significantly higher expression of *MYH2*, therefore more type IIA fibres than the CC/II (p ≤ 0.001) and TT/NN (p ≤ 0.001) cell lines. Fibre type proportions, expressed as a percentage of total, for each fibre type are displayed in Fig. 2A and shown in Table 1. These results are consistent with previous observations from *ex vivo* samples of skeletal muscle tissue of Thoroughbreds and their respective genotypes [55,58].

**Table 1:** **Fibre type proportions in immortalised cell lines.** Data expressed as % of total (±SEM) n = 3 per genotype.

	I	IIA	IIX
**CC/II**	1.4 (±0.6)	23.0 (±1.8)	75.6 (±2.4)
**CT/IN**	4.7 (±0.2)	45.8 (±2.3)	49.5 (±2.5)
**TT/NN**	33.4 (±2.5)	33.3 (±1.2)	33.3 (±1.3)

A key feature of the CC/II genotype is the lower expression of *MSTN* mRNA in skeletal muscle with the consequent increase in muscle mass [84] and fast-twitch fibre type [54,55,57]. Here, consistent with previous *ex vivo* skeletal muscle sample analysis [54], *MSTN* gene expression was significantly higher in the TT/NN cell line compared to the CT/IN (p ≤ 0.001) and the CC/II (p ≤ 0.001) cell lines (Fig. 2B).

Considering these results, which were consistent with the previous *ex vivo* findings, we anticipated that we would observe variation in mitochondrial abundance among the 3 cell lines. With a higher proportion of slow twitch muscle fibres compared to fast twitch fibres, the results were as expected, the TT/NN cell line displayed significantly higher citrate synthase (a robust marker of mitochondrial abundance) activity, compared to the CT/IN (p ≤ 0.01) and the CC/II (p ≤ 0.05) cell lines (Fig. 2C). This pattern directly reflects previous findings in skeletal muscle homogenates from TT/NN horses that had significantly greater mitochondrial abundance (determined by citrate synthase activity and mitochondrial DNA content) when compared to CC/II skeletal muscle homogenates [55].

### 3.3 In vitro *CoQ*_*10*_*supplementation increases complex I + III activity*

Previously we found that *ex vivo* skeletal muscle homogenates from TT/NN horses had a lower combined mitochondrial complex I + III activity, indicative of a lower endogenous CoQ_10_ level, when compared to CC/II skeletal muscle homogenates [55]. This lower mitochondrial complex I + III activity level in the TT/NN homogenates could be restored *in vitro* by the addition of exogenous CoQ_10_ to *ex vivo* skeletal muscle homogenates. In a separate study, exogenous CoQ_10_ supplementation *in vivo* to a cohort of horses showed that oral CoQ_10_ supplementation increased complex I + III activity in *ex vivo* skeletal muscle of Thoroughbreds [59].

Here, to test the hypothesis that CoQ_10_ supplementation *in vitro* would increase cellular complex I + III activity (indicating increased CoQ_10_) in skeletal muscle, we added, exogenous CoQ_10_ to the three conditionally immortalised cell lines *in vitro* at a concentration of 5 μM for 24 h. Cells were then lysed and CoQ_10_ content was measured indirectly using the combined mitochondrial complex I + III enzyme activity. We observed that in the untreated cells complex I + III activity was significantly higher in the CC/II cells as compared to the CT/IN (p ≤ 0.05) and TT/NN (p ≤ 0.01) cells (Fig. 2D), an observation consistent with previous *ex vivo* muscle homogenate data [55]. Furthermore, addition of exogenous CoQ_10_ (5 μM for 24 h) increased complex I + III enzyme activity significantly in all three genotype cell lines (p ≤ 0.05), a result that is also in agreement with previously published results [59].

### 3.4 Cellular respiration and oxidative metabolism of conditionally immortalised skeletal muscle cell lines

Having established that these novel immortal cell lines exhibit phenotypic and metabolic characteristics equivalent to *ex vivo* and *in vivo* tissue samples we have determined that the conditionally immortalised TT/NN, CT/IN and the CC/II cell lines authentically reflect the skeletal muscle phenotype of Thoroughbred horses with each genotype. Therefore, these three cell lines are a valid *in vitro* model of variable *MSTN* genotype skeletal muscle and may be used for further characterisation of horse skeletal muscle function.

Therefore, to compliment the mitochondrial complex activity assays, we performed a comparison of cellular oxygen consumption between the three cell lines. Differences in mitochondrial abundance and function are reflected in cellular oxygen consumption [85]. We measured basal cellular oxygen consumption using the Seahorse XFp extracellular flux analyser using three selective mitochondrial inhibitors to assess different aspects of mitochondrial function 1) rate of proton leak (oligomycin to inhibit ATP synthase) 2) maximal respiratory capacity (carbonyl cyanide-p-trifluoromethoxyphenylhydrazone (FCCP) to uncouple the mitochondria) and 3) non-mitochondrial respiration rate (antimycin A/rotenone which inhibit complex III and I respectively, effectively inhibiting all mitochondrial respiration) [85,86].

**Fig. 3:**
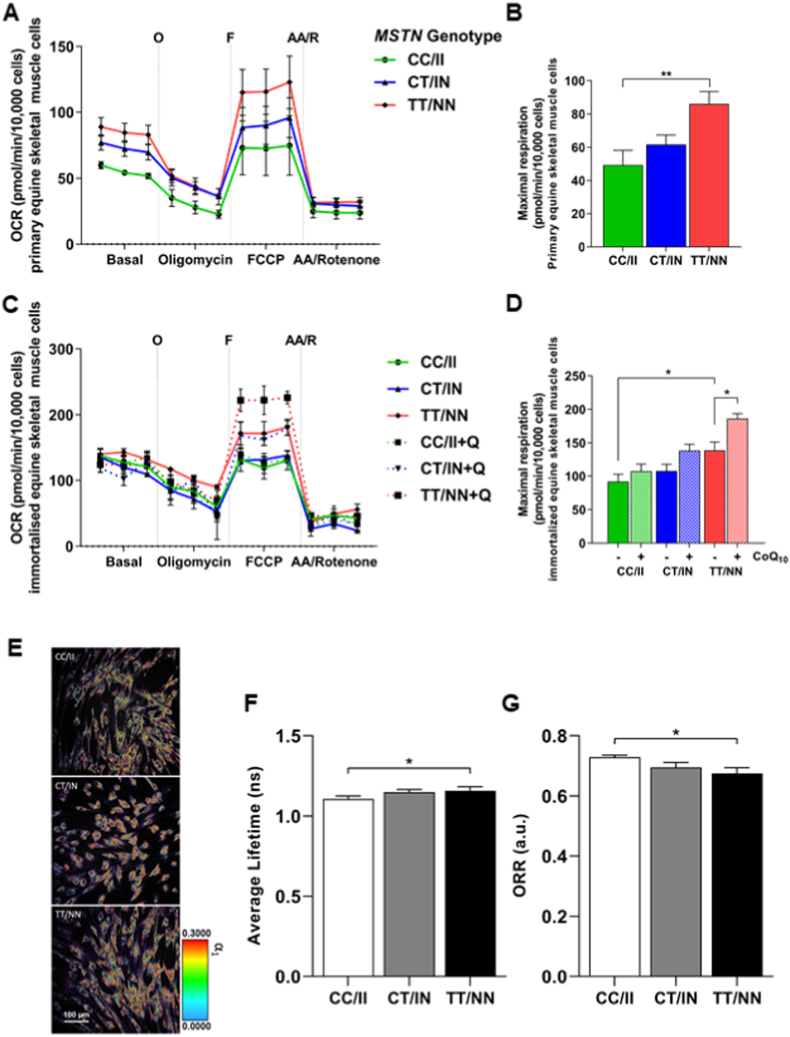
**Cellular respiration and oxidative metabolism:** (A–D) Cellular oxygen consumption rates (OCR), determined by Seahorse XFp Extracellular Flux analyser. Mitochondrial inhibitors/uncouplers were added sequentially during the experiment in the following concentrations: oligomycin 1 μM (O), FCCP 1 μM (F) and antimycin A/rotenone 0.5 μM (AA/R). *MSTN* genotypes; CC/II (green), CT/IN (blue) and TT/NN (red); (A) OCR of primary equine skeletal muscle cells; (B) maximal respiration (maximum rate measurement after FCCP injection) – (non-mitochondrial respiration after AA/R injection) primary equine skeletal muscle cells; (C) OCR of immortalised equine skeletal muscle cells; - and + CoQ_10_ (5 μM for 24 h); (D) maximal respiration immortalised equine skeletal muscle cells, - (plain bars) and + CoQ_10_ (5 μM for 24 h) (dotted bars). (E–G) FLIM analysis of the immortalised cell lines (NAD(P)H-FLIM imaging; marker of oxidative metabolism); (E) average lifetime (ns); (F) optical redox ratio (a.u.); (G) NAD(P)H-FLIM images, each image is representative of 3 independent experiments. Results presented with mean ± SEM (N = 3 per genotype, performed in triplicate technical replicates), p-values where shown indicate significance as measured by; a one-way (B, E, F) or two-way (D) ANOVA with a Tukeys multiple comparisons test * = p ≤ 0.05, ** = p ≤ 0.01, *** = p ≤ 0.001. (For interpretation of the references to colour in this figure legend, the reader is referred to the Web version of this article.)

Comparison of the cell lines in both primary (Fig. 3A/B) and immortalised (Fig. 3C/D) conditions, showed no significant difference in basal, proton leak or non-mitochondrial oxygen consumption rates among the three genotypes. However, after the addition of the uncoupler FCCP, primary TT/NN cells reached a greater maximal respiration rate than the primary CC/II cells (p ≤ 0.05). A similar profile was observed in the immortalised cell lines, with the TT/NN cell line also attaining a higher maximal oxygen consumption rate compared to the CC/II (p ≤ 0.05) cell line. These oxygen consumption results likely reflect the greater mitochondrial abundance in TT/NN compared to CC/II cell lines.

Finally, to further assess the oxidative status of the cells, we performed fluorescence lifetime imaging (FLIM) analysis, which is a non-invasive technique to measure NAD(P)H fluorescence lifetimes and respective fractions in cells (Fig. 3E). Values used to calculate the average fluorescence lifetime (τ_avg_) of NAD(P)H are shown in Supplementary Table 1 (additional file 3). An increase of τ_avg_ values is indicative of a cellular metabolic preference for the oxidative phosphorylation pathway [[87], [88], [89]]. Our results (Fig. 3F) showed that the TT/NN cell line was significantly more oxidative than the CC/II cell line (p ≤ 0.05), an observation consistent with the mitochondrial abundance and function results.

In addition to the NAD(P)H fluorescence lifetimes, the optical redox ratio (ORR) of the different cell lines was calculated. ORR is a ratio of the fluorescence intensity of FAD^+^ and NAD(P)H which provides insight on the cell oxidative and metabolic state [87,90,91]. FAD^+^ is a fluorescence metabolic co-factor that during the Krebs cycle is reduced to FADH recovered by hydrogen transfer at the Complex II of the electron transport chain. NAD(P)H is formed during the Krebs cycle or earlier steps of glycolysis and oxidised to NAD(P)^+^ at Complex I [89,92,93].

Our results show a statistically significant increase in the ORR of CC/II cell line when compared with TT/NN cell line. However, no statistical significance was found when compared with CT/IN (Fig. 3G). The increase in the ORR can be justified by a shift towards a decrease of the NAD(P)H pool while maintaining or increasing FAD^+^ availability. Our previous results (Fig. 2C) show an increase in citrate synthase activity in the TT/NN cell line which is likely reflective of a higher abundance of mitochondria and NAD(P)H molecules [72]. In addition, complex I + III activity is reduced in the TT/NN cell line (Fig. 2D) which decreases the consumption of NAD(P)H by the electron transport chain. The conjugation of both results indicates an increase of NAD(P)H availably resulting in a reduction of the ORR (Fig. 3G), leading to the conclusion that the TT/NN cell line is more oxidative than the CC/II cell line as measured by FLIM analysis (Fig. 3E/F/G).

### 3.5 Maximal mitochondrial respiratory capacity of skeletal muscle cell lines was increased by CoQ_10_ supplementation

Since we previously demonstrated that oral supplementation with CoQ_10_ *in vivo* and exogenous CoQ_10_ during *in vitro* experiments improved mitochondrial complex I + III activity in *ex vivo* skeletal muscle samples from TT/NN horses, we tested whether supplementation of CoQ_10_ to the TT/NN cell line improved *in vitro* mitochondrial function. The addition of CoQ_10_ (5 μM for 24 h) had no significant effect on any cell line for basal, proton leak or non-mitochondrial respiration rates (Fig. 3C/D). However, at maximal respiration capacity, the TT/NN cell line, when treated with CoQ_10_, attained a higher maximal oxygen consumption rate than untreated cells (p ≤ 0.05). We therefore conclude that CoQ_10_ augmented the maximal mitochondrial electron transport chain capacity in the TT/NN cells. CoQ_10_ has previously been proposed as a potential supplement to improve exercise capacity, aerobic power and recovery after exercise [[94], [95], [96], [97], [98]]. Here, we provide *in vitro* evidence indicating that CoQ_10_ supplementation improves the mitochondrial respiratory capacity of skeletal muscle cells.

## 4 Conclusions

We have established conditionally immortalised equine skeletal muscle cell lines as *in vitro* cell models. Characterisation of these cell lines in terms of *MSTN* gene expression, muscle fibre type and metabolic function, confirmed that they mirror *ex vivo* skeletal muscle tissue phenotypes from equivalent genotype horses (*e.g.* fast-twitch fibres, low mitochondrial abundance and low *myostatin* expression in CC/II genotype) and are representative of Thoroughbred horse skeletal muscle. Employing this *in vitro* model we assessed cellular oxygen consumption, previously limited due to the logistical challenges of obtaining *ex vivo* muscle specimens, and we demonstrated that addition of CoQ_10_ improves mitochondrial function in these cell lines, consistent with previous *in vivo* studies and *ex vivo* studies. Furthermore, we demonstrated that CoQ_10_ can increase the maximal mitochondrial respiration capacity in skeletal muscle cells. We maintain that these conditionally immortalised cell lines are representative of their original *ex vivo* skeletal muscle source and are reliable models of Thoroughbred skeletal muscle. These cell lines will be a valuable resource for future equine skeletal muscle research and may be applied to a range of investigations relevant to the Thoroughbred industry, from supplement analysis to uses in gene doping or detection of prohibited substances.

## Ethics approval and consent to participate

All samples were obtained post-mortem from animals euthanized for non-research purposes. Owners consent was given.

## Consent for publication

Not applicable.

## Availability of data and materials

The datasets generated and analysed during the study are available from the corresponding author on reasonable request.

## Credit author statement

**Mary F Rooney:** conceptualization, methodology, validation, formal analysis, investigation, resources, writing - original draft, writing - review & editing, visualization, supervision, project administration, funding acquisition. **Nuno Neto:** Investigation, writing - review & editing. **Michael Monaghan:** resources, writing - review & editing. **Emmeline W Hill:** data curation, writing - review & editing. **Richard K Porter:** supervision, writing - review & editing.

## Funding

This work was supported by 10.13039/501100001588Enterprise Ireland (IP/2016/0503 awarded to RKP and IP/2019/0768 awarded to MFR). NN was supported by 10.13039/501100001637Trinity College Dublin, Provost's PhD Award. The TCD FLIM core unit is supported by 10.13039/501100001602Science Foundation Ireland (SFI) Infrastructure Programme (16/RI/3403 awarded to MM).

## Declaration of competing interest

EWH is a shareholder in and Chief Scientific Officer of Plusvital Ltd., an equine nutrition and genetic testing company. Plusvital Ltd. has been granted a licence for commercial use of the data that is contained within multiple granted patents and patent applications including (patent reference numbers): EP2352850, JP5667057, US8771943, AU2009290452, NZ591711, US9249470 and US2016215335. EWH is named on these patents. MFR, NGBN, MGM and RKP declare that they have no competing interests. Other than EWH, Plusvital Ltd. played no role in the study design, data collection and analysis, decision to publish, or preparation of the manuscript.
