# Supplementary material for: Conditionally immortalised equine skeletal muscle cell lines for in vitro analysis
Source: Biochem Biophys Rep. 2022 Dec 5;33:101391. doi: 10.1016/j.bbrep.2022.101391 (PMC9727643; doi:10.1016/j.bbrep.2022.101391)
Supplement: Multimedia component 1 [file mmc1.pdf]

Additional File 1: Figure S1: Equine myoblast immortalisation GFP control image, SV40T protein and SV40T PCR confirmation: (A) Primary equine myoblasts from three *MSTN* (CC/II, CT/IN, TT/NN) genotype Thoroughbreds were transfected with a GFP conjugated Lenti-SV40 vector to evaluate transfection efficiency. Cells were grown and passaged as required for 14 days after transfection. Green indicates GFP positive (i.e. positive transfection) cells. Cells were imaged on an Olympus IX81 inverted microscope at 10X. Scale bar (white) indicates 10 µm. (B) Protein extracted from conditionally immortalised myoblast cells was analysed for SV40T protein expression by immunoblot. The SV40T protein is positively expressed in the 3 cell types (CC/II, CT/IN and TT/NN) and untransfected equine myoblasts (UT) do not express the protein as expected. Immunoblots shown are representative of at least 3 independent experiments. (C) DNA was extracted from conditionally immortalised myoblast cells and a PCR to detect SV40T DNA was performed; 1: CC/II, 2: CT/IN, 3: TT/NN, 4: SV40T plasmid (positive control), 5: Untransfected primary equine skeletal muscle cell DNA. (B and C) The images have been cropped in this figure; full immunoblot/gel images are presented in Additional File 5.

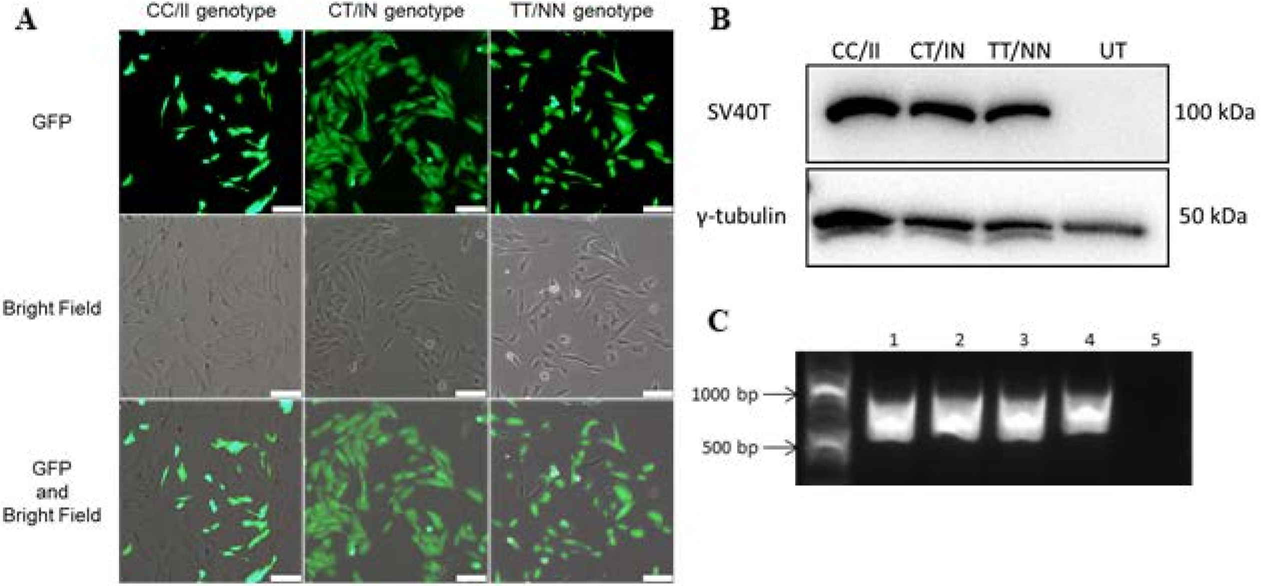
A
CC/II genotype
CT/IN genotype
TT/NN genotype
GFP
Bright Field
GFP and Bright Field
B
CC/II
CT/IN
TT/NN
UT
SV40T
100 kDa
γ-tubulin
50 kDa
C
1
2
3
4
5
1000 bp
500 bp

Additional File 2: Figure S2: Desmin and sarcomeric myosin expression in 3T3L1 and C2C12 cells: Immunofluorescence images of 3T3L1 cells (non-myogenic control), undifferentiated C2C12 cells (myogenic control) and differentiated C2C12 myotubes (myogenic and differentiation control). Green, desmin (myogenic marker)/sarcomeric myosin (myosin) (differentiation marker); blue, nuclei. Images were take using a Leica SP8 confocal microscope at 20X. Scale bar (white) indicates 50 µM. Each image is representative of at least 3 independent experiments.

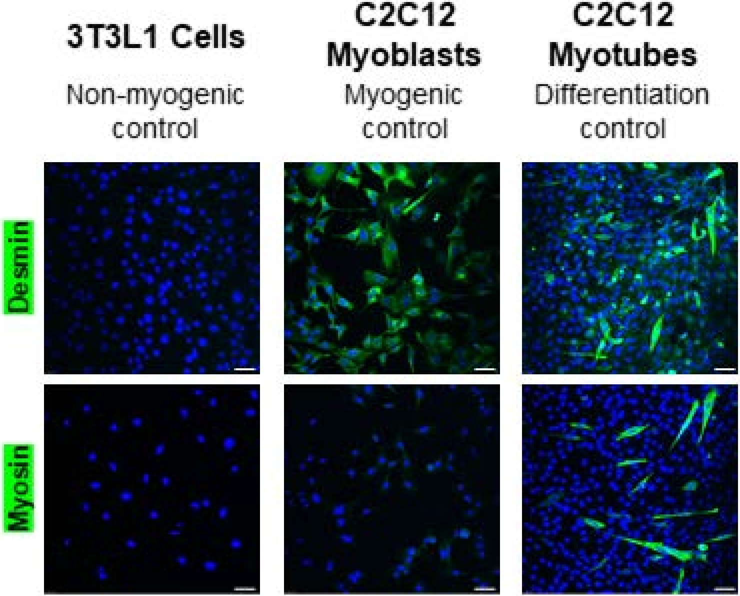
3T3L1 Cells
Non-myogenic control
C2C12 Myoblasts
Myogenic control
C2C12 Myotubes
Differentiation control
Desmin
Myosin

Additional File 3: Table S1: Summary of produced NAD(P)H-FLIM imaging data for different cell types.

Table S1: Summary of produced NAD(P)H-FLIM imaging data for different cell types.

Cell Type	Average lifetime NAD(P)H ± SD, ns	Protein-bound NAD(P)H lifetime ± SD, ns	Fraction of bound NAD(P)H ± SD	Free NAD(P)H lifetime ± SD, ns
CC	1.107±0.018	2.898±0.042	0.254±0.004	0.499±0.009
CT	1.149±0.017	2.884±0.028	0.267±0.008	0.516±0.004
TT	1.158±0.025	2.956±0.052	0.266±0.007	0.507±0.009

Additional File 4: Supplementary methods: Methods pertaining to data shown in additional file 1.

Supplementary methods

GFP imaging

Primary equine myoblasts from three *MSTN* (CC/II, CT/IN, TT/NN) genotype Thoroughbreds were transfected with a GFP conjugated Lenti-SV40 vector to evaluate transfection efficiency. Cells were grown and passaged as required for 14 days after transfection in 6 well plates. Cells were imaged *in situ* using an Olympus IX81 inverted microscope at 10X magnification. Green fluorescence indicated GFP positive (i.e. positive transfection) cells.

Detection of SV40T by PCR

DNA was extracted from cell pellets using a proteinase K and DTT digestion in TENT buffer (10 mM Tris-HCL; pH 8.0, 10 mM EDTA, 100 mM NaCl, 2% Tween-20) followed by a phenol/chloroform/isoamyl alcohol (24:25:1) separation and ethanol precipitation. A polymerase chain reaction (PCR)-based assay was used to determine the presence of the SV40T gene in cell samples. The primer sequences were as follows: 5’ AGCCTGTAGAACCAAACATT 3' (forward) and 5’ CTGCTGACTCTCAACATTCT 3' (reverse). The PCR mix consisted of GoTaq Hot start master mix (Promega, WI), primers (forward and reverse) at final concentrations of 0.5 µM each and extracted DNA. The PCR conditions were, as follows; initial denaturation at 95 °C for 5 minutes, followed by 40 cycles of 95 °C for 30 seconds, 50 °C for 40 seconds and 72 °C for 1 minute, followed by a final elongation step of 72 °C for 5 minutes. The resulting amplification products were electrophoresed on a 2% agarose gel and the presence of a 792 bp band indicted the SV40T gene.

Detection of SV40T by immunoblot

Protein was extracted from skeletal muscle cell pellets using RIPA buffer (50 mM Tris-HCL; pH 8.0, 150 mM NaCl, 0.1% SDS, 1% NP-40, 0.5% sodium deoxycholate, protease inhibitors). An aliquot was used to perform a BCA assay to determine the protein concentration and the remainder was used for SDS-PAGE and immunoblotting. Proteins were resolved by Sodium dodecyl sulphate-polyacrylamide gel electrophoresis (SDS-PAGE) as per the method of Laemmli et al. [1] and transferred to polyvinylidene difluoride (PVDF) membranes (Immobolin-P^{SQ}; Sigma-Aldrich) using a semi-dry transfer system (Hoefer Inc.). Membranes were blocked by incubation in TBS-Tween (TBS supplemented with 0.1 % (v/v) Tween) (TBST) supplemented with 5 % (w/v) non-fat dry milk powder for 1 hour at room temperature. Blots were then incubated in polyclonal primary antibody (mouse anti-SV40 Tag antibody, Catalogue no. G202, ABM at 1:1000 or mouse anti-GAPDH antibody (loading control), Catalogue no. CB1001, Calbiochem at 1:5000) diluted in fresh blocking buffer overnight at 4 °C. The secondary antibody used was an anti-mouse horse-radish peroxidase-linked (HRP) antibody (Promega) diluted in blocking buffer at a 1:1000 dilution. The secondary antibody was incubated with the membrane for 1 hour at room temperature before blots were developed using an enhanced chemiluminescence (ECL) detection system (Millipore Immobilon ECL Western Blotting Substrate) for detecting horseradish peroxidase labelled antibody, by means of the HRP catalysed oxidation of luminol under alkaline conditions and the results were visualised by ChemiDoc (Bio-Rad) computerised system and Image Lab software. Densitometry analysis was performed using Image Lab Software Analysis function.

1. Laemmli UK. Cleavage of structural proteins during the assembly of the head of bacteriophage t4. Nature. 1970;227(5259):680-5. 10.1038/227680a0.

Additional File 5: Full gel and immunoblot pertaining to Figure S1.

SV40T

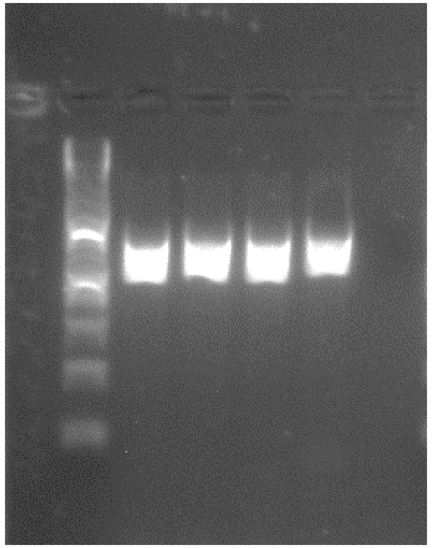

Lane 1: Ladder
Lane 2: CC/II
Lane 3: CT/IN
Lane 4: TT/NN
Lane 5: + (SV40TtsA58 vector)
Lane 6: - (H2O)

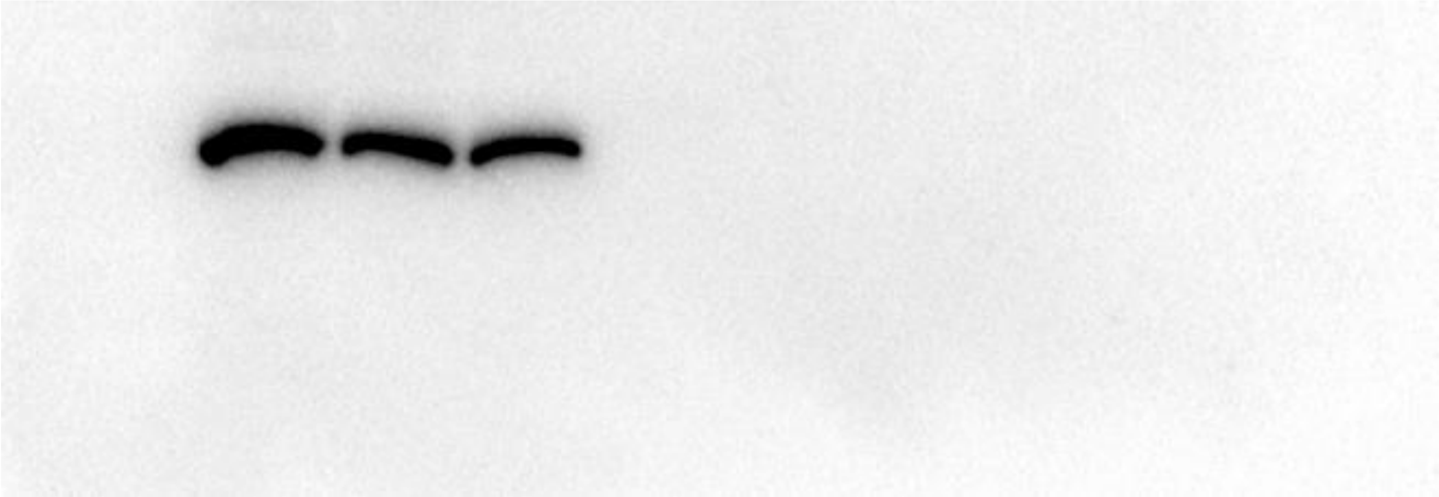

SV40T

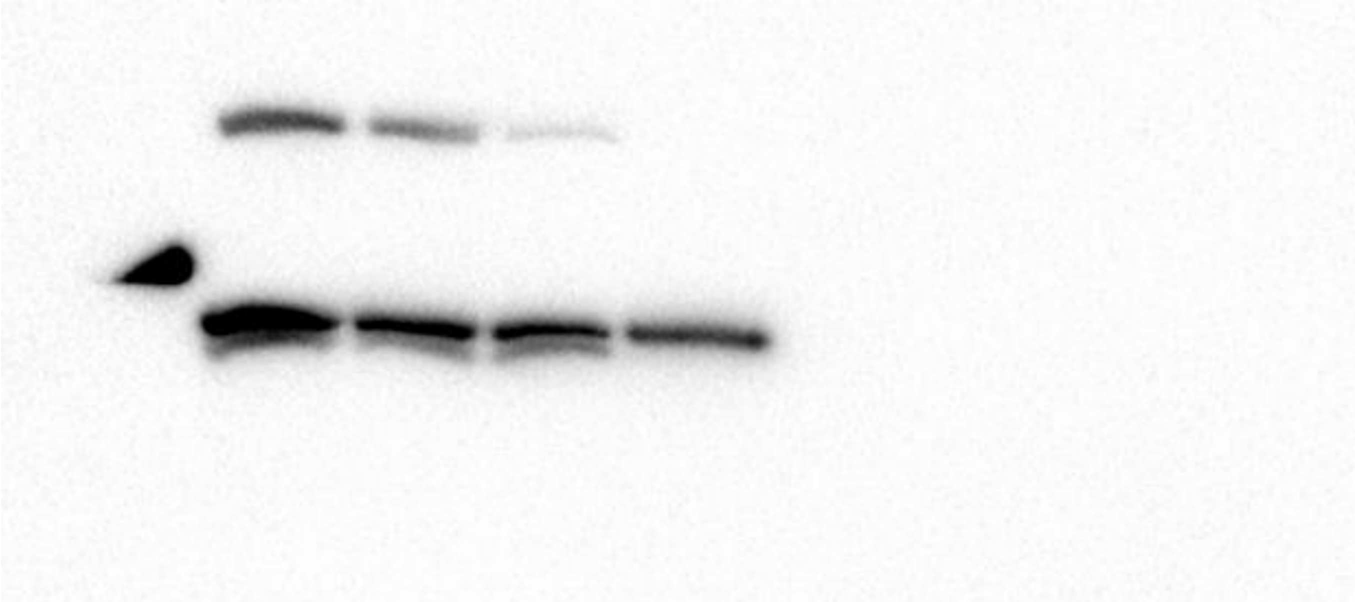

γ-tubulin
